# Isopropyl 2-(5-fluoro-3-methyl­sulfinyl-1-benzofuran-2-yl)acetate

**DOI:** 10.1107/S1600536809037003

**Published:** 2009-09-19

**Authors:** Hong Dae Choi, Pil Ja Seo, Byeng Wha Son, Uk Lee

**Affiliations:** a Department of Chemistry, Dongeui University, San 24 Kaya-dong Busanjin-gu, Busan 614-714, Republic of Korea; b Department of Chemistry, Pukyong National University, 599-1 Daeyeon 3-dong, Nam-gu, Busan 608-737, Republic of Korea

## Abstract

In the title compound, C_14_H_15_FO_4_S, the O atom and the methyl group of the methyl­sulfinyl substituent are located on opposite sides of the plane of the benzofuran fragment which is essentially planar with a mean deviation of 0.008 (1) Å from its least-squares plane. The crystal structure stabilized by three different inter­molecular non-classical C—H⋯O hydrogen bonds. The crystal structure also exhibits aromatic π–π inter­actions between the benzene rings of adjacent benzofuran ring systems [centroid–centroid distance = 3.688 (2) Å]

## Related literature

For the crystal structures of similar alkyl 2-(5-fluoro-3-methyl­sulfinyl-1-benzofuran-2-yl) acetate derivatives, see: Choi *et al.* (2009**a* ▶,b* ▶). For the pharmacological activity of benzofuran compounds, see: Howlett *et al.* (1999 ▶); Twyman & Allsop (1999 ▶).
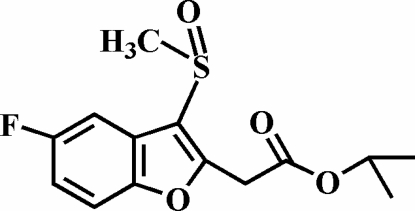


## Experimental

### 

#### Crystal data

C_14_H_15_FO_4_S
*M* *_r_* = 298.32
Monoclinic, 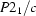

*a* = 11.6332 (6) Å
*b* = 14.9522 (7) Å
*c* = 8.2333 (4) Å
β = 102.277 (1)°
*V* = 1399.36 (12) Å^3^
*Z* = 4
Mo *K*α radiation
μ = 0.25 mm^−1^
*T* = 173 K
0.25 × 0.20 × 0.16 mm

#### Data collection

Bruker SMART CCD diffractometer
Absorption correction: multi-scan (*SADABS*; Sheldrick, 2000 ▶) *T*_min_ = 0.940, *T*_max_ = 0.961
12229 measured reflections
3173 independent reflections
2476 reflections with *I* > 2σ(*I*)
*R*_int_ = 0.043

#### Refinement

*R*[*F* ^2^ > 2σ(*F* ^2^)] = 0.036
*wR*(*F* ^2^) = 0.098
*S* = 1.09
3173 reflections
182 parameters
H-atom parameters constrained
Δρ_max_ = 0.39 e Å^−3^
Δρ_min_ = −0.39 e Å^−3^

### 

Data collection: *SMART* (Bruker, 2001 ▶); cell refinement: *SAINT* (Bruker, 2001 ▶); data reduction: *SAINT*; program(s) used to solve structure: *SHELXS97* (Sheldrick, 2008 ▶); program(s) used to refine structure: *SHELXL97* (Sheldrick, 2008 ▶); molecular graphics: *ORTEP-3* (Farrugia, 1997 ▶) and *DIAMOND* (Brandenburg, 1998 ▶); software used to prepare material for publication: *SHELXL97*.

## Supplementary Material

Crystal structure: contains datablocks I. DOI: 10.1107/S1600536809037003/bq2158sup1.cif

Structure factors: contains datablocks I. DOI: 10.1107/S1600536809037003/bq2158Isup2.hkl

Additional supplementary materials:  crystallographic information; 3D view; checkCIF report

## Figures and Tables

**Table 1:** Hydrogen-bond geometry (Å, °)

*D*—H⋯*A*	*D*—H	H⋯*A*	*D*⋯*A*	*D*—H⋯*A*
C5—H5⋯O3^i^	0.93	2.50	3.370 (2)	155
C6—H6⋯O2^ii^	0.93	2.54	3.369 (2)	149
C9—H9*B*⋯O4^iii^	0.97	2.26	3.228 (2)	176

Symmetry codes: (i) 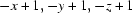; (ii) 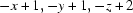; (iii) 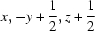.

**Fig. 1.**
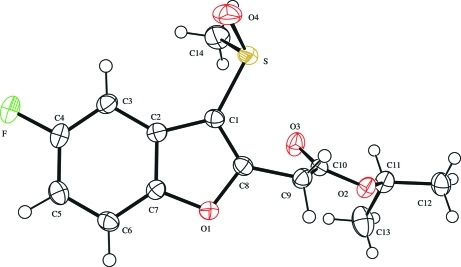
The molecular structure of the title compound with the atom numbering scheme. Displacement ellipsoids are drawn at the 50% probability level.

**Fig. 2.**
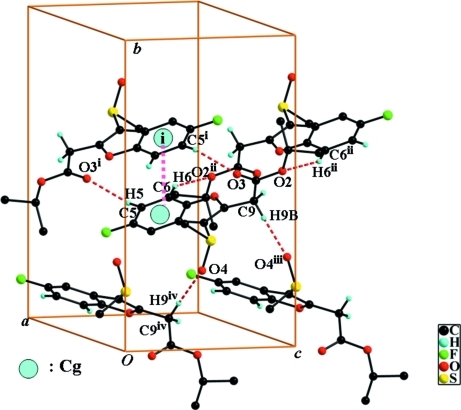
The C—H···O and π···π interactions (dotted lines) in the title compound. Cg denotes the benzene ring centroid. [Symmetry codes: (i) - x + 1, - y + 1, - z + 1; (ii) - x + 1, - y + 1, - z + 2; (iii) x, - y, z + 1/2; (iv) x, - y + 1/2, z - 1/2.]

## supplementary crystallographic information

### Comment

Molecules involving benzofuran skeleton have attracted particular interest in view of their biological and pharmacological properties (Howlett *et al.*, 1999; Twyman & Allsop, 1999). As a part of our ongoing studies of the effect of side chain substituents on the solid state structures of alkyl 2-(5-fluoro-3-methylsulfinyl-1-benzofuran-2-yl) acetate analogues (Choi *et al.*, 2009*a,b*), we report the crystal structure of the title compound (Fig. 1).

The benzofuran unit is essentially planar, with a mean deviation of 0.008 (1) Å from the least-squares plane defined by the nine constituent atoms. The crystal packing (Fig. 2) is stabilized by three intermolecular non-classical C—H···O hydrogen bonds; the first between an H atom of the benzofuran ring and the oxygen of the C═O unit, with a C5–H5···O3^i^, the second between an H atom of the benzofuran ring and the oxygen of the isopropoxy group, with a C6—H6···O2^ii^, the third between a methylene H atom and the oxygen of the S═O unit, with a C9—H9B···O4^iii^, respectively (Table 1). The crystal packing (Fig. 2) is further stabilized by aromatic π···π interactions between the benzene rings of neighboring molecules, with a Cg···Cg^i^ distance of 3.688 (2) Å (Cg is the centroid of the C2–C7 benzene ring).

### Experimental

77% 3-chloroperoxybenzoic acid (247 mg, 1.1 mmol) was added in small portions to a stirred solution of isopropyl 2-(5-fluoro-3-methylsulfanyl-1-benzofuran-2-yl) acetate (282 mg, 1.0 mmol) in dichloromethane (30 ml) at 273 K. After being stirred for 4 h at room temperature, the mixture was washed with saturated sodium bicarbonate solution and the organic layer was separated, dried over magnesium sulfate, filtered and concentrated in vacuum. The residue was purified by column chromatography (hexane–ethyl acetate, 1:2 v/v) to afford the title compound as a colorless solid [yield 83%, m.p. 391–392 K; *R*_f_ = 0.67 (hexane–ethyl acetate, 1;2 v/v )]. Single crystals suitable for X-ray diffraction were prepared by evaporation of a solution of the title compound in acetone at room temperature. Spectroscopic analysis: EI-MS 298 [M^+^].

### Refinement

All H atoms were geometrically positioned and refined using a riding model, with C—H = 0.93 Å for the aryl, 0.97 Å for the methine, 0.98 Å for the methylene, and 0.96 Å for the methyl H atoms. U_iso_(H) = 1.2U_eq_(C) for the aryl, methine, and methylene H atoms, and 1.5U_eq_(C) for the methyl H atoms.

### Crystal data

**Table:**

C_14_H_15_FO_4_S	*F*(000) = 624
*M**_r_* = 298.32	*D*_x_ = 1.416 Mg m^−^^3^
Monoclinic, *P*2_1_/*c*	Mo *K*α radiation, λ = 0.71073 Å
Hall symbol: -P 2ybc	Cell parameters from 5216 reflections
*a* = 11.6332 (6) Å	θ = 2.3–27.4°
*b* = 14.9522 (7) Å	µ = 0.25 mm^−^^1^
*c* = 8.2333 (4) Å	*T* = 173 K
β = 102.277 (1)°	Block, colorless
*V* = 1399.36 (12) Å^3^	0.25 × 0.20 × 0.16 mm
*Z* = 4	

### Data collection

**Table:**

Bruker SMART CCD diffractometer	3173 independent reflections
Radiation source: fine-focus sealed tube	2476 reflections with *I* > 2σ(*I*)
graphite	*R*_int_ = 0.043
Detector resolution: 10.0 pixels mm^-1^	θ_max_ = 27.5°, θ_min_ = 1.8°
φ and ω scans	*h* = −14→15
Absorption correction: multi-scan (*SADABS*; Sheldrick, 2000)	*k* = −19→19
*T*_min_ = 0.940, *T*_max_ = 0.961	*l* = −10→10
12229 measured reflections	

### Refinement

**Table:**

Refinement on *F*^2^	Primary atom site location: structure-invariant direct methods
Least-squares matrix: full	Secondary atom site location: difference Fourier map
*R*[*F*^2^ > 2σ(*F*^2^)] = 0.036	Hydrogen site location: inferred from neighbouring sites
*wR*(*F*^2^) = 0.098	H-atom parameters constrained
*S* = 1.09	*w* = 1/[σ^2^(*F*_o_^2^) + (0.0423*P*)^2^ + 0.5417*P*] where *P* = (*F*_o_^2^ + 2*F*_c_^2^)/3
3173 reflections	(Δ/σ)_max_ = 0.001
182 parameters	Δρ_max_ = 0.39 e Å^−^^3^
0 restraints	Δρ_min_ = −0.39 e Å^−^^3^

### Special details

**Table:**

Geometry. All esds (except the esd in the dihedral angle between two l.s. planes) are estimated using the full covariance matrix. The cell esds are taken into account individually in the estimation of esds in distances, angles and torsion angles; correlations between esds in cell parameters are only used when they are defined by crystal symmetry. An approximate (isotropic) treatment of cell esds is used for estimating esds involving l.s. planes.
Refinement. Refinement of F^2^ against ALL reflections. The weighted R-factor wR and goodness of fit S are based on F^2^, conventional R-factors R are based on F, with F set to zero for negative F^2^. The threshold expression of F^2^ > 2sigma(F^2^) is used only for calculating R-factors(gt) etc. and is not relevant to the choice of reflections for refinement. R-factors based on F^2^ are statistically about twice as large as those based on F, and R- factors based on ALL data will be even larger.

### Fractional atomic coordinates and isotropic or equivalent isotropic displacement parameters (Å^2^)

**Table:**

	*x*	*y*	*z*	*U*_iso_*/*U*_eq_	
S	0.16889 (4)	0.32823 (3)	0.59899 (5)	0.02546 (13)	
F	0.53377 (11)	0.32123 (8)	0.19202 (14)	0.0427 (3)	
O1	0.47612 (10)	0.43326 (8)	0.79555 (14)	0.0242 (3)	
O2	0.19830 (10)	0.54133 (8)	1.03615 (14)	0.0264 (3)	
O3	0.17889 (11)	0.53806 (9)	0.75830 (15)	0.0329 (3)	
O4	0.17181 (12)	0.23235 (9)	0.54858 (18)	0.0393 (3)	
C1	0.31410 (14)	0.36896 (11)	0.6411 (2)	0.0222 (3)	
C2	0.40529 (14)	0.36553 (11)	0.5455 (2)	0.0222 (3)	
C3	0.41420 (16)	0.33514 (12)	0.3875 (2)	0.0264 (4)	
H3	0.3516	0.3078	0.3155	0.032*	
C4	0.52168 (16)	0.34834 (12)	0.3458 (2)	0.0289 (4)	
C5	0.61801 (16)	0.38849 (12)	0.4473 (2)	0.0284 (4)	
H5	0.6880	0.3950	0.4111	0.034*	
C6	0.60937 (14)	0.41885 (12)	0.6032 (2)	0.0257 (4)	
H6	0.6722	0.4464	0.6744	0.031*	
C7	0.50247 (14)	0.40604 (11)	0.6470 (2)	0.0224 (3)	
C8	0.36106 (14)	0.40933 (11)	0.7872 (2)	0.0229 (4)	
C9	0.31082 (15)	0.43478 (12)	0.9330 (2)	0.0252 (4)	
H9A	0.3743	0.4524	1.0239	0.030*	
H9B	0.2725	0.3831	0.9689	0.030*	
C10	0.22306 (14)	0.51050 (12)	0.8949 (2)	0.0234 (4)	
C11	0.11677 (16)	0.61749 (12)	1.0234 (2)	0.0298 (4)	
H11	0.0555	0.6121	0.9218	0.036*	
C12	0.06204 (17)	0.61224 (13)	1.1728 (2)	0.0340 (4)	
H12A	0.0191	0.5572	1.1699	0.051*	
H12B	0.0094	0.6618	1.1718	0.051*	
H12C	0.1226	0.6143	1.2721	0.051*	
C13	0.1857 (2)	0.70278 (14)	1.0176 (3)	0.0504 (6)	
H13A	0.2183	0.7028	0.9199	0.076*	
H13B	0.2482	0.7064	1.1146	0.076*	
H13C	0.1344	0.7533	1.0147	0.076*	
C14	0.11297 (18)	0.39058 (15)	0.4135 (2)	0.0397 (5)	
H14A	0.1529	0.3725	0.3282	0.060*	
H14B	0.1258	0.4532	0.4356	0.060*	
H14C	0.0302	0.3794	0.3773	0.060*	

### Atomic displacement parameters (Å^2^)

**Table:**

	*U*^11^	*U*^22^	*U*^33^	*U*^12^	*U*^13^	*U*^23^
S	0.0205 (2)	0.0281 (2)	0.0274 (2)	−0.00173 (17)	0.00402 (16)	−0.00285 (18)
F	0.0481 (7)	0.0526 (8)	0.0320 (6)	−0.0002 (6)	0.0189 (5)	−0.0125 (5)
O1	0.0211 (6)	0.0286 (6)	0.0221 (6)	−0.0011 (5)	0.0031 (5)	−0.0028 (5)
O2	0.0283 (6)	0.0295 (7)	0.0213 (6)	0.0066 (5)	0.0046 (5)	−0.0010 (5)
O3	0.0323 (7)	0.0439 (8)	0.0226 (6)	0.0096 (6)	0.0062 (5)	0.0042 (6)
O4	0.0339 (7)	0.0291 (7)	0.0529 (9)	−0.0065 (6)	0.0048 (6)	−0.0116 (6)
C1	0.0203 (8)	0.0202 (8)	0.0253 (8)	0.0012 (6)	0.0030 (6)	0.0000 (7)
C2	0.0222 (8)	0.0196 (8)	0.0241 (8)	0.0022 (6)	0.0033 (6)	0.0002 (7)
C3	0.0281 (9)	0.0243 (9)	0.0258 (8)	0.0002 (7)	0.0037 (7)	−0.0046 (7)
C4	0.0359 (10)	0.0275 (9)	0.0254 (9)	0.0052 (8)	0.0113 (8)	−0.0022 (7)
C5	0.0256 (9)	0.0279 (9)	0.0339 (9)	0.0044 (7)	0.0108 (7)	0.0028 (8)
C6	0.0200 (8)	0.0263 (9)	0.0298 (9)	0.0015 (7)	0.0029 (7)	0.0005 (7)
C7	0.0241 (8)	0.0213 (8)	0.0217 (8)	0.0028 (7)	0.0042 (6)	0.0000 (6)
C8	0.0198 (8)	0.0233 (8)	0.0251 (8)	0.0009 (6)	0.0037 (6)	0.0026 (7)
C9	0.0263 (9)	0.0283 (9)	0.0205 (8)	0.0009 (7)	0.0037 (7)	0.0005 (7)
C10	0.0215 (8)	0.0274 (9)	0.0214 (8)	−0.0046 (7)	0.0052 (6)	−0.0015 (7)
C11	0.0299 (9)	0.0320 (10)	0.0269 (9)	0.0098 (8)	0.0049 (7)	0.0021 (8)
C12	0.0301 (9)	0.0382 (11)	0.0359 (10)	0.0079 (8)	0.0119 (8)	0.0019 (9)
C13	0.0648 (15)	0.0322 (11)	0.0636 (15)	0.0043 (10)	0.0345 (13)	0.0087 (11)
C14	0.0302 (10)	0.0517 (13)	0.0329 (10)	0.0040 (9)	−0.0030 (8)	0.0067 (9)

### Geometric parameters (Å, °)

**Table:**

S—O4	1.4949 (14)	C6—C7	1.380 (2)
S—C1	1.7595 (17)	C6—H6	0.9300
S—C14	1.7894 (19)	C8—C9	1.491 (2)
F—C4	1.365 (2)	C9—C10	1.512 (2)
O1—C8	1.3731 (19)	C9—H9A	0.9700
O1—C7	1.384 (2)	C9—H9B	0.9700
O2—C10	1.338 (2)	C11—C12	1.502 (2)
O2—C11	1.472 (2)	C11—C13	1.513 (3)
O3—C10	1.205 (2)	C11—H11	0.9800
C1—C8	1.353 (2)	C12—H12A	0.9600
C1—C2	1.450 (2)	C12—H12B	0.9600
C2—C7	1.394 (2)	C12—H12C	0.9600
C2—C3	1.402 (2)	C13—H13A	0.9600
C3—C4	1.380 (2)	C13—H13B	0.9600
C3—H3	0.9300	C13—H13C	0.9600
C4—C5	1.384 (3)	C14—H14A	0.9600
C5—C6	1.385 (3)	C14—H14B	0.9600
C5—H5	0.9300	C14—H14C	0.9600
			
O4—S—C1	108.00 (8)	C10—C9—H9A	109.0
O4—S—C14	106.75 (10)	C8—C9—H9B	109.0
C1—S—C14	98.47 (9)	C10—C9—H9B	109.0
C8—O1—C7	106.03 (12)	H9A—C9—H9B	107.8
C10—O2—C11	117.35 (13)	O3—C10—O2	124.43 (16)
C8—C1—C2	107.15 (14)	O3—C10—C9	125.76 (15)
C8—C1—S	121.23 (13)	O2—C10—C9	109.79 (14)
C2—C1—S	131.57 (13)	O2—C11—C12	106.27 (14)
C7—C2—C3	119.14 (15)	O2—C11—C13	108.45 (16)
C7—C2—C1	104.64 (14)	C12—C11—C13	112.95 (17)
C3—C2—C1	136.21 (16)	O2—C11—H11	109.7
C4—C3—C2	115.75 (16)	C12—C11—H11	109.7
C4—C3—H3	122.1	C13—C11—H11	109.7
C2—C3—H3	122.1	C11—C12—H12A	109.5
F—C4—C3	117.80 (16)	C11—C12—H12B	109.5
F—C4—C5	117.32 (16)	H12A—C12—H12B	109.5
C3—C4—C5	124.87 (16)	C11—C12—H12C	109.5
C4—C5—C6	119.52 (16)	H12A—C12—H12C	109.5
C4—C5—H5	120.2	H12B—C12—H12C	109.5
C6—C5—H5	120.2	C11—C13—H13A	109.5
C7—C6—C5	116.35 (16)	C11—C13—H13B	109.5
C7—C6—H6	121.8	H13A—C13—H13B	109.5
C5—C6—H6	121.8	C11—C13—H13C	109.5
C6—C7—O1	124.87 (15)	H13A—C13—H13C	109.5
C6—C7—C2	124.37 (16)	H13B—C13—H13C	109.5
O1—C7—C2	110.75 (14)	S—C14—H14A	109.5
C1—C8—O1	111.43 (14)	S—C14—H14B	109.5
C1—C8—C9	132.54 (16)	H14A—C14—H14B	109.5
O1—C8—C9	115.98 (14)	S—C14—H14C	109.5
C8—C9—C10	112.79 (14)	H14A—C14—H14C	109.5
C8—C9—H9A	109.0	H14B—C14—H14C	109.5
			
O4—S—C1—C8	−126.67 (15)	C3—C2—C7—C6	0.0 (3)
C14—S—C1—C8	122.54 (15)	C1—C2—C7—C6	−178.66 (16)
O4—S—C1—C2	50.55 (18)	C3—C2—C7—O1	178.64 (14)
C14—S—C1—C2	−60.23 (18)	C1—C2—C7—O1	0.01 (18)
C8—C1—C2—C7	−0.17 (18)	C2—C1—C8—O1	0.27 (19)
S—C1—C2—C7	−177.69 (14)	S—C1—C8—O1	178.10 (11)
C8—C1—C2—C3	−178.44 (19)	C2—C1—C8—C9	177.57 (17)
S—C1—C2—C3	4.0 (3)	S—C1—C8—C9	−4.6 (3)
C7—C2—C3—C4	0.1 (2)	C7—O1—C8—C1	−0.26 (18)
C1—C2—C3—C4	178.20 (18)	C7—O1—C8—C9	−178.05 (14)
C2—C3—C4—F	−178.87 (15)	C1—C8—C9—C10	−69.4 (2)
C2—C3—C4—C5	0.0 (3)	O1—C8—C9—C10	107.82 (16)
F—C4—C5—C6	178.67 (16)	C11—O2—C10—O3	−3.7 (2)
C3—C4—C5—C6	−0.2 (3)	C11—O2—C10—C9	178.11 (14)
C4—C5—C6—C7	0.3 (3)	C8—C9—C10—O3	13.5 (2)
C5—C6—C7—O1	−178.66 (15)	C8—C9—C10—O2	−168.35 (14)
C5—C6—C7—C2	−0.2 (3)	C10—O2—C11—C12	154.84 (15)
C8—O1—C7—C6	178.81 (16)	C10—O2—C11—C13	−83.45 (19)
C8—O1—C7—C2	0.15 (18)		

### Hydrogen-bond geometry (Å, °)

**Table:**

*D*—H···*A*	*D*—H	H···*A*	*D*···*A*	*D*—H···*A*
C5—H5···O3^i^	0.93	2.50	3.370 (2)	155
C6—H6···O2^ii^	0.93	2.54	3.369 (2)	149
C9—H9B···O4^iii^	0.97	2.26	3.228 (2)	176

Symmetry codes: (i) −*x*+1, −*y*+1, −*z*+1; (ii) −*x*+1, −*y*+1, −*z*+2; (iii) *x*, −*y*+1/2, *z*+1/2.
